# Non-imported malaria in Italy: paradigmatic approaches and public health implications following an unusual cluster of cases in 2017

**DOI:** 10.1186/s12889-020-08748-9

**Published:** 2020-06-05

**Authors:** Daniela Boccolini, Michela Menegon, Marco Di Luca, Luciano Toma, Francesco Severini, Gianluca Marucci, Stefania D’Amato, Anna Caraglia, Francesco Paolo Maraglino, Giovanni Rezza, Roberto Romi, Luigi Gradoni, Carlo Severini, The Italian Malaria Surveillance Group, Silva Franchini, Marino Migazzi, Roberta Ferranti, Francesco Castelli, Lina Tomasoni, Annapaola Rizzoli, Daniele Arnoldi, Gioia Capelli, Fabrizio Montarsi, Simone Martini, Zeno Bisoffi, Francesca Perandin, Chiara Piubelli, Zeno Pasquini, Benedetta Canovari, Barbara Pieretti, Massimo Agostini, Giorgia Angeloni, Elisa Antognini, Stefano Gavaudan, Michele Conversano, Francesco Desiante, Rosita Cipriani, Roberta Cambria, Ettore Franco, Angelo Pellegrino, Giovanni Battista Buccoliero, Maria Assunta Cafiero, Donato Antonio Raele, Domenico Galante, Pier Angela Ferrero, Anna Bertorello, Paolo Roberto, Andrea Mosca, Sandra Roversi, Laura Gardenghi, Roberto Cagarelli, Giovanna Mattei, Claudio Gualanduzzi

**Affiliations:** 1 Dipartimento Malattie Infettive, Istituto Superiore di Sanità, Viale Regina Elena 299, 00161 Rome, Italy; 2 Ministero della Salute, Direzione Generale della Prevenzione Sanitari, Ufficio 5 – Prevenzione delle Malattie Trasmissibili e Profilassi Internazionale, Rome, Italy; 3 Ministero della Salute, Direzione Generale della Prevenzione Sanitaria, Ufficio 1 - Affari generali e segreteria tecnico-organizzativa, Rome, Italy

**Keywords:** Non-imported malaria, Hospital-acquired infection, Cryptic case, *Plasmodium falciparum*, *Plasmodium ovale* spp., Genotyping, Malaria vector, *Anopheles labranchiae*

## Abstract

**Background:**

The European region achieved interruption of malaria transmission during the 1970s. Since then, malaria control programs were replaced by surveillance systems in order to prevent possible re-emergence of this disease. Sporadic cases of non-imported malaria were recorded in several European countries in the past decade and locally transmitted outbreaks of *Plasmodium vivax*, most probably supported by *Anopheles sacharovi*, have been repeatedly reported from Greece since 2009. The possibility of locally-transmitted malaria has been extensively studied in Italy where the former malaria vector *An. labranchiae* survived the control campaign which led to malaria elimination. In this study, we present paradigmatic cases that occurred during a 2017 unusual cluster, which caused strong concern in public opinion and were carefully investigated after the implementation of the updated malaria surveillance system.

**Methods:**

For suspected locally-transmitted malaria cases, alerts to Ministry of Health (MoH) and the National Institute of Health (ISS) were mandated by the Local Health Services (LHS). Epidemiological investigations on the transmission modes and the identification of possible infection’s source were carried out by LHS, MoH and ISS. Entomological investigations were implemented locally for all suspected locally-transmitted cases that occurred in periods suitable to anopheline activity. Molecular diagnosis by nested-PCR for the five human *Plasmodium* species was performed to support microscopic diagnosis. In addition, genotyping of *P. falciparum* isolate was carried out to investigate putative sources of infection and transmission modalities.

**Results:**

In 2017, a cluster of seven non-imported cases was recorded from August through October. Among them, *P. ovale curtisi* was responsible of one case whereas six cases were caused by *P. falciparum*. Two cases were proved to be nosocomial while the other five were recorded as cryptic at the end of epidemiological investigations.

**Conclusions:**

The epidemiological evidence shows that the locally acquired events are sporadic, often remain unresolved and classified as cryptic ones despite investigative efforts. The “cluster” of seven non-imported cases that occurred in 2017 in different regions of Italy therefore represents a conscious alert that should lead us to maintain a constant level of surveillance in a former malaria endemic country.

## Background

Malaria still represents the main imported infectious disease in non-endemic countries, strongly related to the increase in travelers and migratory flows to and from endemic areas [1–3]. Malaria was eliminated from the European region around 1970. Since then, malaria control programs were replaced by surveillance systems to carefully monitor disease importation and prevent its possible re-emergence [4, 5]. Actually, a potential risk for local transmission still lies in the widespread endemic presence of putative mosquito vectors all over the region, mainly belonging to the *Anopheles maculipennis* complex (Diptera: Culicidae) [6].

In the past decade, sporadic cases of non-imported malaria were recorded in several European countries such as Germany, France, Spain and Malta [7–12], and locally transmitted outbreaks of *Plasmodium vivax*, most probably supported by *Anopheles sacharovi*, have been repeatedly reported from Greece since 2009 [5, 13–15]. The possibility of locally-transmitted malaria has been extensively studied in Italy [16, 17], where the former malaria vector *Anopheles labranchiae* survived, and resurged after the malaria elimination campaign [18]. Presently, this species is recorded from scattered foci of coastal rural areas of central and southern Italy where it may reach high abundance in the warm season [6, 19]. Populations of *An. labranchiae* have strongly been suspected to be responsible for autochthonous *P. vivax* transmission after malaria elimination [20, 21], whereas it appears that this species has a low susceptibility to the afro-tropical *Plasmodium falciparum* according to experimental and epidemiological evidence [17, 22, 23].

The Greek situation has renewed malaria concerns by the Italian health authorities, and in December 2016 the Ministry of Health (MoH) and the National Institute of Health (ISS) have released updated guidelines for malaria surveillance. They include a more efficient notification flow of diagnosed cases, with the aim of improving the interaction between Local Health Services (LHS) and the Reference centers, MoH and ISS, both for the surveillance of imported malaria cases and the early detection, followed by prompt control intervention, in case of suspected locally-acquired events [24].

In this study, we present paradigmatic non-imported cases that occurred during a 2017 cluster, which caused strong concern in public opinion and were carefully investigated after the implementation of the updated malaria surveillance system. Surveillance activities carried out to decipher putative infection sources of non-imported cases are described. Findings obtained from the investigations as well as major constraints encountered will be discussed in view of further improvement of the current malaria surveillance system in Italy.

## Methods

### Notification, microscopic confirmation and epidemiological investigation

For suspected locally-transmitted malaria cases that occurred after the implementation of the new malaria surveillance system, alerts to MoH and ISS were activated by LHS. All cases were rapidly notified to both reference institutions by electronic reporting forms, and blood slide samples were sent to ISS for diagnosis confirmation. Epidemiological investigations on the transmission modes and the identification of possible infection’s source were carried out by LHS, MoH and ISS where appropriate.

### Entomological investigation and mosquito processing

Entomological investigations were implemented by LHS for all suspected locally-transmitted cases that occurred in periods suitable to anopheline mosquito activity, targeting both adult and larval stages. The entomological study included inspections for mosquito presence in both the patient’s residence and surrounding areas. In addition, all sites of possible mosquito-bite exposure previously frequented by patients were also inspected. Traps were positioned within a radius of at least 500 m from the home of the case as well as in all the other surveyed areas. Resting females were collected in indoor sites (mainly animal shelters) using manual or battery-powered aspirators or dry ice-CDC light traps. In potential breeding sites (i.e. irrigation and drainage canals, streams, large ponds and other permanent water collections), larvae were collected using a 500 ml standard dipper [17, 24].

The mosquito specimens collected were firstly morphologically identified to select *Anopheles* species [25, 26]. Molecular species identification of all *maculipennis* specimens was performed by multiplex PCR, or ITS2 gene sequencing in case of doubtful multiplex PCR results [27, 28].

### Molecular analysis of *Plasmodium* spp.

Molecular diagnosis by nested-PCR for the five plasmodial species, *P. falciparum*, *P. vivax*, *Plasmodium malariae*, *Plasmodium ovale curtisi* and *Plasmodium ovale wallikeri*, was performed occasionally to support microscopic diagnosis [29, 30]. In addition, genotyping of *P. falciparum* isolates was carried out to investigate putative sources of infection and transmission modalities. DNA was extracted from blood samples (tubes or smears) by using PureLink Genomic DNA Kit (Invitrogen), according to the manufacturer’s instruction. The genotyping was performed by amplification of four highly polymorphic markers, i.e. merozoite surface protein 1 (*Pfmsp1*, block II) and its allelic subfamilies (K1, RO33, MAD20); merozoite surface protein 2 (*Pfmsp2*) and its allelic subfamilies (3D7, FC27) [31]; glutamate-rich protein (GLURP, region II) [32]; and circumsporozoite protein (CS, central region) [33, 34]. When needed, genetic analysis of additional polymorphic targets was employed, such as histidine-rich protein 2 (*Pfhrp2*) and histidine-rich protein 3 (*Pfhrp3*) [35, 36]. The amplification products were analyzed using a high-resolution capillary electrophoresis (QIAxcel Advanced system, Qiagen) suitable for fragment size analysis. PCR products were sent to Eurofins Genomics Company (Germany) for sequencing. The obtained sequences were compiled and analyzed by Accelrys Gene software (Additional files 1 and 2 in Supplementary Information).

### Malaria case definitions

An index malaria case was the patient who came first to the attention of the LHS [10]. Based on findings from the epidemiological investigation, non-imported malaria cases were classified as induced, introduced or cryptic according the WHO terminology. In particular, induced malaria includes any parenteral contagion, either iatrogenic (or nosocomial), post-transfusion and post-transplantation infection; in a non-endemic area, introduced malaria indicates transmission by local competent vector previously infected on an imported case; all cases with inconclusive investigations, i.e. the source of infection remains undefined, are considered cryptic [37–39].

For studies of this type, approval from an Ethical Committee and formal consent are not required because patients’ information, which were anonymized, was derived from the National Surveillance System for malaria [24].

## Results

### Malaria situation in Italy before the implementation of the new surveillance system

In the period 2000–2016, 12,032 malaria cases were diagnosed in Italy, with a yearly average of 715 cases, ranging from a minimum of 575 to a maximum of 977. About 20% of them have occurred in Italian travelers. In the same period, 20 cases (0.17%) were recorded as non-imported ones, having no history of recent travels to endemic areas (Table 1). Particularly, 11 cases were classified as induced (by blood transfusion, organ transplantation or hospital-devices contamination) [40, 41]; 7 were considered cryptic; 2 were strongly suspected to be introduced [21]. The latter cases consisted of *P. vivax*-infected individuals who resided in formerly malaria-endemic territories of Latium (August 2009) and Calabria regions (September 2011), respectively.

**Table 1:** Annual incidence and references of non-imported malaria cases reported in Italy in 2000–2018

Years	N. of non-imported cases	*Plasmodium* species	Event definition	Reference	% (Total cases)
2000	1	*P. falciparum*	Induced (nosocomial)	40	0.10 (977)
2001	0	–	–		0.00 (888)
2002	0	–	–		0.00 (733)
2003	1	*P. falciparum*	Induced (suspected)		0.15 (681)
2004	2	*P. falciparum*	Induced (post-transplant)		0.30 (673)
		*P. falciparum*	Cryptic		
2005	1	*P. malariae*	Induced (transfusional)	41	0.16 (637)
2006	0	–	–		0.0 (630)
2007	2	*P. falciparum*	Induced (suspected)		0.35 (575)
		*P. falciparum*	Induced (suspected)		
2008	2	*P. vivax*	Cryptic		0.34 (583)
		*P. falciparum*	Induced (suspected)		
2009	2	*P. vivax*	Introduced (suspected)	21	0.31 (636)
		*P. falciparum*	Induced (suspected)		
2010	2	*P. ovale*	Cryptic		0.29 (700)
		*P. malariae*	Induced (suspected)		
2011	1	*P. vivax*	Introduced (suspected)	21	0.14 (701)
2012	1	*P. falciparum*	Cryptic		0.16 (642)
2013	2	*P. malariae*	Induced (suspected)		0.30 (677)
		*P. falciparum*	Induced (suspected)		
2014	2	*P. falciparum*	Cryptic		0.28 (705)
		*P. malariae*	Cryptic		
2015	1	*P. malariae*	Cryptic		0.14 (706)
2016	0	–	–		0.00 (888)
**TOTAL**	**20**				**0.17 (12,032)**
2017	7	*P. ovale*	Cryptic	43,44	0.84 (831)
		*P. falciparum*	Induced (nosocomial)	10,11	
		*P. falciparum*	Induced (nosocomial)	11	
		*P. falciparum*	Cryptic	42,45	
		*P. falciparum*	Cryptic	42,45	
		*P. falciparum*	Cryptic	42,45	
		*P. falciparum*	Cryptic	42,45	
2018	3	*P. falciparum*	Cryptic	51	0.42 (722)
		*P. falciparum*	Cryptic		
		*P. falciparum*	Cryptic		
**TOTAL**	**10**				**0.64 (1553)**

### Imported and non-imported malaria cases in Italy since 2017 (Table 1; Fig. 1)

In 2017, out of 831 cases documented in Italy, a worrying number of seven non-imported cases was recorded from August through October. Specifically, for these patients (0.84% of the total number of cases, (Table 1) histories excluded travels to endemic areas, blood transfusion or organ transplantation. Among them, *P. ovale* was responsible for one case recorded in Pesaro, central Italy; six cases were infected by *P. falciparum*, one of whom (a fatal case) was from Trento (north Italy), four from Taranto (south Italy) and one from Florence (central Italy) [10, 11, 42–45].

**Fig. 1:**
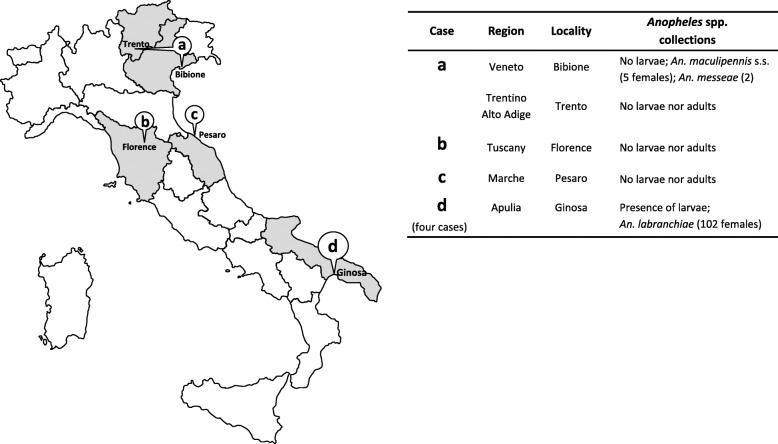
Geographical distribution of non-imported malaria cases occurred in 2017 in Italy (grey regions), and associated findings from entomological investigations targeting *Anopheles* vectors species (The map shown in Fig. 1 is an original drawing created by the authors and has not been taken from other sources)

### Investigations on cases eventually resulted as induced (nosocomial)

#### Fatal malaria case from Trento

On 2 September 2017, an Italian girl under five with no history of travel to a malaria-endemic country was admitted to a hospital in Trento with subsequent diagnosis of *P. falciparum* malaria. Due to her severe condition, she was transferred to the tropical diseases reference Centre in Brescia, Lombardy, and despite treatment with intravenous artesunate she died on 4 September. She had been previously admitted (on August 13) in Portogruaro hospital, the nearest to Bibione, a town on the Adriatic coast of northwestern Italy where she was on holiday, and there she was diagnosed with diabetes mellitus. On August 16 she was transferred to a hospital of Trento, where she was discharged on August 21. On August 31 she came back to the emergency room of the hospital, for a febrile episode, and she was diagnosed with pharyngitis; then, she was admitted again on 2 September in severe conditions and received a diagnosis of malaria. An epidemiological investigation carried out after the child’s death, identified in the Hospital the presence of four members of one family affected by malaria, mother and her three children, two girls and one boy, who recently returned from Burkina Faso. In particular, the two young sisters, infected with *P. falciparum* were hospitalized in the same ward of the Italian girl, during her stay in the Trento hospital from August 16 to 21, while, their brother and mother were hospitalized in the same hospital but in different wards.

Entomological investigations for presence of *Anopheles* spp. were performed in areas surrounding the resort of Bibione, where the child had spent her holidays from August 1-13, as well as in Trento, in areas around the family’s home and the hospital compound. In Bibione, nine sites were inspected for adult mosquitoes, mainly consisting of animal farms. Six potential larval breeding sites, represented by drinking troughs, artificial containers, ditches and irrigation canals, and a drainage channel, were also inspected. Species belonging to Culicinae subfamily were mainly collected (*Aedes albopictus, Culex pipiens* complex*, Ochlerotatus caspius*). However, in a buffalo farm and in a horse breeding farm six adult specimens of *An. maculipennis* s.l. were collected by manual aspirators, whereas one specimen was caught inside a powerhouse. The seven specimens, molecularly identified, were five *Anopheles maculipennis* s.s. and two *Anopheles messeae* [46]. The collections performed in the urban sites of Trento did not yield anopheline mosquitoes, despite previous reports of *Anopheles plumbeus, An. maculipennis* s.l. and *Anopheles claviger* s.l. from annual routine mosquito monitoring activities [47].

Parasites from the blood of the Italian child (index case) and the four Burkinabe family members were confirmed microscopically and molecularly to belong to *P. falciparum*. Genotyping of the isolates using polymorphic markers resulted as follows: in all five samples, one clone attributable to the subfamily *Pfmsp1*-K1 and one clone attributable to the *Pfmsp1*-RO33 family were identified. *Pfmsp1*-RO33 showed both size and sequence identity with the index case and all the members of the Burkinabe family. Moreover, *Pfmsp1*-K1 showed size and sequence identity with the index case and the two sisters. This latter result excluded the brother and mother as a source of infection (Table 2). CS size of the index case and both sisters was identical, however the CS sequence was only identical between the index case and the older sister. For *Pfhrp2*, two clones having identical size were recorded in the index case and the older sister. For *Pfhrp3*, fragment size and sequence identity between the index case and the older sister was demonstrated, but both the size and the sequence were different from the younger sister. This last result excluded the younger sister as a source of infection of the Italian case, leaving the older Burkinabe sister as the most probable parasitological index case (Table 2).

**Table 2:** Epidemiological and parasite molecular features of 5 malaria cases hospitalized in Trento. The *Plasmodium falciparum* polymorphic markers analysed showed complete concordance between the isolates 1 (putative induced; the reference case) and 2 (imported)

Patient	Epidemiological characteristics of the patient				Comparison among the patients for five polymorphic molecular markers from *P. falciparum* isolates				
	Country of origin	Gender (family member)	Onset of symptoms	Hospitalization period	MSP1-K1	MSP1-RO33	CS	HRP2	HRP3
1 (putative induced)	Italy	Female	28/08/2017	16-20/08/2017	*	*	*	*	*
2 (imported)	Burkina Faso	Female (old sister)	14/08/2017	16-21/08/2017	*	*	*	*	*
3 (imported)	Burkina Faso	Female (young sister)	19/08/2017	20-24/08/2017	*	*	**	***	****
4 (imported)	Burkina Faso	Male (brother)	14/08/2017	17-21/08/2017	****	*	n.a.	n.a.	n.a.
5 (imported)	Burkina Faso	Female (mother)	18/08/2017	19-24/08/2017	****	*	n.a.	n.a.	n.a.

* Size and nucleotide sequence identity; ** Size identity but discordance in nucleotide sequence; *** Size identity in only one of the identified clones;

**** Discordance in size and nucleotide sequence; *n.a.* not applicable

#### Malaria case from Florence

In October 2017, an Italian teenage boy was admitted to the emergency room of a hospital in Florence (Tuscany Region, Central Italy). The first time he was admitted to emergency room on October 13 and then hospitalized until October 19; he showed respiratory distress, headache, and vomiting, but he was apyretic. The second access to the emergency room was on 28 October, for fever episodes that started on October 26, and the third one on October 31 due to the persistence of the fever, when he was hospitalized.

The clinical picture appeared severe, and the patient received a blood transfusion on November 6. On November 16, to screen for further infectious diseases, he was also tested by PCR for malaria with unexpectedly positive results for *P. falciparum*, with a parasitemia of 1%. Treatment with intravenous artesunate and quinine hydrochloride was administered for 2 days. On the second day of treatment the boy was apyretic with complete remission of symptoms, and parasitaemia index decrease to 0.4%. On 20 November, oral therapy started with piperaquine and dihydroartemisinin; a parasitaemia of 0.01% was observed. Of interest, hematological analyses disclosed a minor degree of thalassemia trait, which probably saved the patient’s life considering the delay from putative infection diagnosis (see below) and successful treatment.

The boy and his parents did not report travels to countries endemic for malaria. To rule out the possibility that the Italian patient became infected by the blood transfusion received on 6 November, a *Plasmodium* PCR was performed on two samples obtained prior to that date, i.e. a peripheral blood sample collected on October 31 and a bone marrow aspirate collected on November 2. Both samples were positive for *P. falciparum*.

An epidemiological investigation revealed an imported malaria case in another teen ager’s boy, who was admitted in the same hospital with suspected severe malaria on October 13, reporting recent travel to Senegal. The two young boys were registered in the same emergency room on October 13 and both hospitalized in the same ward, the imported case from the 13th to 27th of October, and the index case, as mentioned above, from the 13th to 19th of October.

Entomological investigation was mainly limited to indoor collections performed in the hospital compound and the surrounding area, considering that the index case was diagnosed in late autumn, i.e. a period unsuitable for the life cycle of anopheline mosquitoes in natural environments of Central Italy. Only Culicinae species (*Ae. albopictus*, *Cx. pipiens* complex*, Culiseta longiareolata*) were collected.

**Fig. 2:**
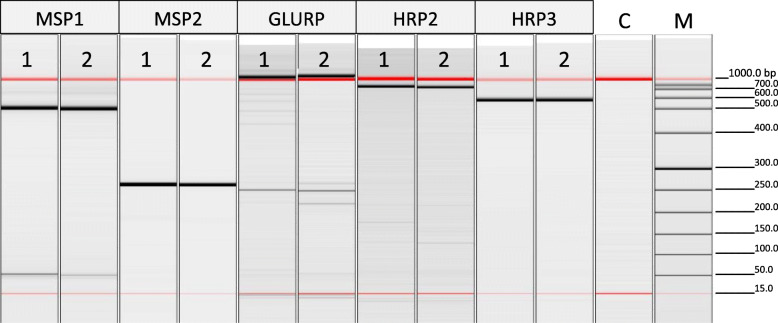
Capillary electrophoresis image showing PCR amplified products of *Plasmodium falciparum* DNA isolated from two malaria cases hospitalized in Florence. (1) Putative induced case; (2) imported case; (C) no DNA; (M) DNA marker. All five polymorphic markers analyzed showed complete size concordance between the two isolates

Parasites from the blood of the Italian (index case) and Senegalese boys were confirmed microscopically and molecularly to be *P. falciparum*. Genotyping of the isolates using polymorphic markers showed full size and sequence identity of amplicons from *Pfmsp1*-K1, *Pfmsp2*-FC27, GLURP (region II), *Pfhrp2*, and *Pfhrp3* (Table 3; Fig. 2). The parasitological index case was therefore considered to be the Senegalese boy.

**Table 3:** Epidemiological and plasmodial molecular features of 2 malaria cases hospitalized in Florence. All 5 polymorphic markers analysed showed complete concordance between the isolates 1 (putative induced; the reference case) and 2 (imported)

Patient	Epidemiological characteristics of the patient				Comparison among the patients for five polymorphic molecular markers from *P. falciparum* isolates				
	Country of origin	Gender	Onset of symptoms	Hospitalization period	MSP1-K1	MSP2-FC27	GLURP	HRP2	HRP3
1 (putative induced)	Italy	Male	26/10/2017	13-19/10/2017	*	*	*	*	*
2 (imported)	Senegal	Male	08/10/2017	13-27/10/2017	*	*	*	*	*

*Size and nucleotide sequence identity

### Investigations on cases eventually reported as cryptic

#### Malaria case from Pesaro

On 18 August 2017, a woman in her 60’s was admitted to the hospital emergency room of Pesaro, an Adriatic seaside town, with fever and shivers that appeared every other day for 1 week. She had never left Italy and did not report recent contacts with people who travelled abroad. Suspecting a hematological disease, a blood smear was performed. Unexpectedly, malaria parasites with morphological traits suggesting of *P. vivax/P. ovale* spp*.* were detected. Moreover, a rapid diagnostic malaria test (RDT - BinaxNow®, ALERE) for *Plasmodium* antigen detection was positive for non-*falciparum* malaria. The patient was treated with piperaquine/dihydroartemisinin for 3 days with excellent clinical response and full recovery.

An epidemiological investigation to identify potential disease-associated risk factors including mosquito-bite exposure was conducted. Of note, endemic malaria transmission had never been reported in the Pesaro territory in the past; moreover, no cases of imported malaria were documented in the area in the previous months. An entomological survey targeting both adult mosquitoes and larvae of *Anopheles* spp. was carried out in eight sites, including urban places around the patient’s home and the hospital. Only Culicinae larvae and adults (*Ae. albopictus, Cx. pipiens* complex) were collected.

**Fig. 3:**
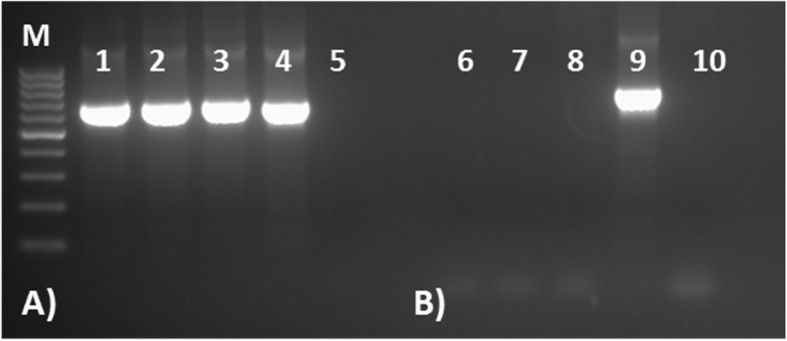
PCR amplification of plasmodial DNA isolated from the non-imported case from Pesaro, targeting sequences discriminating between *Plasmodium ovale curtisi* and *P. ovale wallikeri*. Two pairs of primers were used: first one pair (rOVA1WC-rOVA2WC) is used to amplify both subspecies, and second one pair (rOVA1v-rOVA2v) is specific for *P. ovale wallikeri* diagnosis (Fuehrer et al., 2012). (M) DNA marker; **a** Amplification results using rOVA1WC+ rOVA2WC primers; (1–2) patient sample replicates; (3) *P. ovale curtisi* control DNA; (4) *P. ovale wallikeri* control DNA; (5) negative control (no DNA); **b** Amplification results using rOVA1v + rOVA2v primers; (6-7) patient sample replicates; (8) *P. ovale curtisi* control DNA; (9) *P. ovale wallikeri* control DNA; (10) negative control (no DNA)

The parasite was microscopically confirmed as *P. ovale* spp., and the molecular identification showed that it belonged to the subspecies *P. ovale curtisi* (Fig. 3).

#### Malaria cases from Ginosa, Taranto province

From September 26 through October 2, 2017, four *P. falciparum* malaria cases, three of Moroccan origin and one of Sudanese nationality respectively, aged between 20’s and 30’s years, without declared recent travel history to malaria-endemic countries, were reported with the onset of symptoms in the same week. The first case reported the onset of symptoms on September 20, the last case on September 27. The four patients were admitted to the infectious diseases ward of the same Taranto hospital, complaining high fever, diarrhea and abdominal pain. Splenomegaly and thrombocytopenia were recorded, and a *P. falciparum* malaria was diagnosed in all. The patients, who did not refer any previous malarial attacks, were successfully treated with mefloquine.

An epidemiological investigation showed that two Moroccan patients lived in the same house in a countryside district of Ginosa municipality (Taranto province) together with three people; the third Moroccan patient lived nearby with four people. The Sudanese case lived in the same district and shared home with 6 people of the same nationality. None of the cohabitants declared malaria-like symptoms nor they had travelled abroad during the previous 3 months. None of the four malaria cases had travelled to countries endemic for *P. falciparum* malaria in the previous 3 years. The two younger Moroccan patients had arrived in Italy from Morocco about 3 months before, making a stop in Salerno and moving to Ginosa about 10-20 days before; the other Moroccan patient and the Sudanese declared having been in Italy for over 9 years. All cases referred to be employed as agricultural workers, in the same area or a neighboring commune. They did not report recent hospitalization, surgery or blood transfusion, nor intravenous drug use. The patients denied that they or their roommates had received any type of package or have hosted relatives or friends with luggage from *P. falciparum*-endemic areas. In the face of a putative epidemic cluster of *falciparum* malaria, as a precautionary measure and by order of the National Blood Center, all blood donations by residents or temporary visitors of Ginosa municipality were suspended for 6 months. An entomological survey was carried out in sites of patients’ residence and working places, from October 4 through 10. Adult mosquito specimens were caught in farms and animal shelters from three rural sites and outdoor in one patient’s residence by battery-powered aspirators; larval samplings were made in breeding sites. A total of 102 adults (90 females) and several larvae of *An. maculipennis* s.l. were collected and morphologically and/or molecularly identified as *An. labranchiae*.

Parasites from the blood of the four cases were confirmed microscopically and molecularly to be *P. falciparum*. Genotyping of the isolates showed polyclonal infections as revealed by all polymorphic markers used. Targeted sequences were successfully amplified in all isolates except for the Moroccan case where the GLURP sequence was not amplified. Fragment analysis of the amplified PCR products show, without the need for sequencing, that the four patients were infected by different *P. falciparum* isolates (Table 4), therefore suggesting that this could represent a “false epidemic cluster” (or “pseudo-outbreak”). However, further investigations should be carried out in this area to definitively exclude whether the vector densities recorded could be compatible with the possibility of local transmission events started by infected persons.

**Table 4:** Epidemiological and plasmodial molecular features of 4 malaria cases hospitalized in Taranto. All 9 polymorphic markers analysed showed markedly size discordance among the four isolates

Patient	Epidemiological characteristics of the patient				Comparison among the patients for nine polymorphic molecular markers from *P. falciparum* isolates								
	Country of origin	Gender	Onset of symptoms	Hospitalization period	MSP1	MSP1-K1	MSP1-RO33	MSP1-MAD20	MSP2	MSP2-FC27	GLURP	HRP2	HRP3
1 (cryptic)	Sudan	Male	20/09/2017	30/09/2017	**	**	*	*	**	*	*	**	*
2 (cryptic)	Morocco	Male	22/09/2017	26/09/2017	*	*	*	*	**	*	n.a.	*	**
3 (cryptic)	Morocco	Male	27/09/2017	01/10/2017	*	*	*	n.a.	*	*	*	**	*
4 (cryptic)	Morocco	Male	27/09/2017	02/10/2017	*	*	*	*	*	*	**	*	*

* Size identity; ** Discordance in size; *n.a.* not applicable

## Discussion

Although malaria cases diagnosed in non-endemic countries are mostly represented by imported, travel-related infections, a small proportion of patients not reporting history of travel to endemic areas come down with infection every year. In Italy, non-imported malaria showed a steady trend over the 2000–2016 period accounting for a 0 to 0.35% annual rate and a total rate of 0.17% among all malaria cases [24, 48, 49]. The unusual rate of non-imported cases recorded in 2017 (0.84%) was followed by a lower incidence in 2018 (0.42%) (Table 1). The occurrence of non-imported malaria in countries that are no longer endemic, such as Italy and other Mediterranean countries, may have an important impact on patient management. Being uncommon, these cases can be misdiagnosed or have delayed diagnosis; the consequences of delayed treatment are particularly dangerous in case of infections due to *P. falciparum,* the most life-threatening species. In rare occasions, non-imported cases can occur as local outbreaks limited in space and time, or in full-fledged malaria re-emergence as recently reported in Greece [14, 50].

The occurrence of locally-transmitted cases should be taken into consideration by health professionals and always suspected in patients with unresolved episodes of fever. The first approach by the clinicians should be a timely and accurate anamnesis of the patient, that should include the collection of information about any activity carried out in a relatively recent time before the onset of the disease: workplace and residence (with particular attention to presence of international airport/harbor or stores/markets of products imported from tropical areas); personal relationships (hosted relatives or friends from endemic areas); hospitalization and/or recent surgery, blood transfusions, drug addiction. In this phase of the investigation, all possible modes of transmission should be explored, therefore the detailed history of the patient is a pivotal starting point to address further investigations and, if necessary, to implement appropriate and rapid health measures. Actually, the malaria notification form of the Italian surveillance system, has been devised to assist properly the initial investigation of the suspected locally-transmitted cases [24].

In the case of foreign patients, additional efforts are needed to reduce issues that could make the interview unreliable; language and religious barriers could lead the patient to give limited and incomplete information, thus the collaboration of cultural mediators can be advisable. This probably happened in the case of the four *P. falciparum*-infected patients from Ginosa, three of them being from Morocco and one from Sudan. Their unclear social and working condition could have probably forced these people to omit crucial details about their actual travels in the period preceding the infection, making the investigation inconclusive.

As for the case that occurred in the Italian patient living in Pesaro, despite an extensive epidemiological and entomological investigation, neither the potential source of the infection nor the mode of transmission were identified. In this event, the most probable cause could be ascribed to “luggage malaria”, i.e. caused by infected tropical mosquitoes imported with goods, since this touristic seaside town hosts a great deal of yard sales run by foreigners during summer. With no further evidence and having excluded malaria transmission by local vectors, the case was classified as cryptic [39].

A major impact of cryptic cases on the public health system in the case of unsolved suspicion of malaria introduction (e.g. in Ginosa or, more recently, in coastal Tuscany) [51] (Table 1), concerns the possible application of recommended blood safety measures [52] involving 6-month suspension of blood donation by residents of the affected municipality. This has been the case of the precautionary measures taken in Ginosa following the falciparum malaria cluster.

Since all the mentioned 2017 malaria events have occurred in the season (August–October) corresponding to the period of *An. maculipennis* s.l*.* activity in Italy, it was necessary to trigger entomological surveys around the index case, to establish the presence/abundance of potential malaria vectors in/around the patient’s residential areas, and/or in/around the hospital compound, as well as in all areas of possible mosquito-bite exposure. Evaluation of the extrinsic incubation period was carried out, considering theoretical times for *Plasmodium* spp. development in anopheline mosquitoes (sporogonic cycle), at the mean temperatures of the period considered. A sporogonic cycle of 11-16 days could be estimated, whereas a cumulative incubation period including extrinsic and intrinsic (in-patient) periods, could be estimated as 20-28 days [17]. For all the seven non-imported cases of 2017 the cumulative incubation period was consistent with the local transmission hypothesis, but the presence of potential *Anopheles* vectors was only detected in Bibione and Ginosa. However, the scarce abundance of collected mosquito samples led to consider the involvement of indigenous vectors very unlikely also in these two localities (Fig. 1).

In Italy, anopheline species belong to the *An. maculipennis* complex, which comprises nowadays 5 species not distinguishable morphologically, *Anopheles atroparvus*, *An. labranchiae, An. maculipennis* s.s., *Anopheles melanoon* and *An. messeae* are widely distributed; a sixth species, *An. sacharovi*, has not been recorded in Italy since 1960 [18]. These species show differences in their behavioral patterns and feeding preferences, giving rise to the presence of Italian areas characterized by “anophelism without malaria” [5, 18]. *Anopheles labranchiae* was the main Italian malaria vector in the pre-malaria eradication era, being reported to be highly anthropophilic as compared with other members of the complex. Less diffuse and less abundant species such as *An. claviger*, *An. plumbeus* and *Anopheles superpictus*, have always been considered secondary malaria vectors, mainly due to their peculiar ecology. Of note, *An. plumbeus* was recently suspected to be involved in locally-transmitted malaria in parts of continental Europe [53]. Past and recent experimental data have shown no or very low susceptibility of *An. labranchiae* populations to afro-tropical strains of *P. falciparum* [17, 22, 23]. Nevertheless, it should be considered that genetic traits (i.e. anthropophilic behavior) and local environmental features (temperature, humidity, land use, accessibility to humans) all together represent factors strongly influencing the efficiency of anopheline mosquitoes to transmit malaria [17, 54–56].

After the unusual cluster of non-imported cases that occurred in 2017, three further non-imported cases were registered in Italy in 2018 (Table 1). They were a Moroccan man attending a hospital in Florence in August [51], and two pregnant women from Nigeria and Ghana hospitalized in September (Cuneo) and in October (Modena), respectively. For these cases, no potential infection source nor putative modes of transmission were identified, and language barriers did not allow getting accurate or reliable epidemiological information. In the Modena case, a prolonged *P. falciparum* infection was strongly suspected on both clinical and epidemiological grounds; post-delivery follow up excluded congenital malaria in the newborn. During the entomological investigations carried out around the residence of the Cuneo case, one specimen of *An. plumbeus* was collected. The poor vector density and the low evening temperatures of that period led to the conclusion that the involvement of this species in a local transmission event was very unlikely.

## Conclusions

Epidemiological investigation on non-imported malaria cases in formerly-endemic countries represents a difficult task, as potential risk factors involve necessarily both, those associated with patient’s daily life, including health care supplies, and environmental parameters potentially associated with transmission. Our paradigmatic examples on hospital-related malaria infections have shown that recent advances in *Plasmodium* genetics provided useful tools for molecular epidemiology investigations. On the other hand, serious knowledge gaps were mainly encountered in the entomological risk assessment, because of (i) lack of accurate and updated distribution maps of Italian anopheline mosquitoes, which have much probably extended their geographical range during the past decades owing to environmental and climate changes; (ii) limited knowledge on the susceptibility of current anopheline populations to infection by different imported *Plasmodium* species and geographical populations.

In conclusion, the locally acquired events are sporadic, often remain unresolved and classified as cryptic ones despite efforts in investigation. The epidemiological evidence show that the risk of malaria spread in Italy associated with such events is low, even in the areas considered most vulnerable where it could give rise mainly to isolated cases of *P. vivax*. The unusual “cluster” of seven non-imported cases occurred in 2017 in different regions of Italy should not therefore be considered as an alarming event, but as a conscious alert that should lead us to maintain a high and constant level of surveillance for this disease on a former malaria endemic country. A periodic assessment of receptivity (presence and abundance of anopheline vectors) and vulnerability (presence of human reservoirs of infection) [5] of the Italian territory should be carried out to further improve surveillance and response in case of non-imported events.

## Supplementary information

**Additional file 1.** Nucleotide sequences from the genetic markers analyzed for the molecular investigations of the putative induced malaria case, Trento 1, and the imported malaria cases, Trento 2-5.

**Additional file 2.** Nucleotide sequences from the genetic markers analyzed for the molecular investigations of the putative induced malaria case, Florence 1, and the imported malaria case, Florence 2.

## Data Availability

All data generated or analyzed during this study are included in National Surveillance System for malaria. The data are not publically available, they are hosted by the central organism, by the National Institute of Health (Istituto Superiore di Sanità) and by the Ministry of Health (MoH). Local Health Services (LHS) can formally request anonymized data related to respective Regions of interest and /or the annual epidemiological data of the National Surveillance System, directly to the ISS.

## Abbreviations

MoH: Ministry of Health
ISS: Istituto Superiore di Sanità (National Institute of Health)
LHS: Local Health Services
RDT: Rapid diagnostic test
*Pfmsp1*: *Plasmodium falciparum* merozoite surface protein 1
GLURP: Glutamate-rich protein
CS: Circumsporozoite protein
*Pfmsp2*: *Plasmodium falciparum* merozoite surface protein 2
*Pfhrp3*: *Plasmodium falciparum* histidine Rich protein 3
WHO: World Health Organization
