# Supplementary material for: Non-imported malaria in Italy: paradigmatic approaches and public health implications following an unusual cluster of cases in 2017
Source: BMC Public Health. 2020 Jun 5;20:857. doi: 10.1186/s12889-020-08748-9 (PMC7275312; doi:10.1186/s12889-020-08748-9)
Supplement: Supplementary file 1 — Additional file 1. Nucleotide sequences from the genetic markers analyzed for the molecular investigations of the putative induced malaria case, Trento 1, and the imported malaria cases, Trento 2-5. [file 12889_2020_8748_MOESM1_ESM.docx]

**Additional file 1.** Nucleotide sequences from the genetic markers analyzed for the molecular investigations of the putative induced malaria case, Trento 1, and the imported malaria cases, Trento 2-5.

**Trento 1** PfMSP1- K1 family partial gene sequence

AGTGGTGCAAGTGCTCAAAGTGGTGCAAGTGCTCAAAGTGGTGCAAGTGCTCAAAGTGGTGCAAGTGCTCAAAGTGGTACAAGTGGTCCAAGTGGTCCAAGTGGTACAAGTCCATCATCTCGTTCAAACACTTTACCTCGTTCAAATACTTCATCTG

**Trento 2** PfMSP1- K1 family partial gene sequence

AGTGGTGCAAGTGCTCAAAGTGGTGCAAGTGCTCAAAGTGGTGCAAGTGCTCAAAGTGGTGCAAGTGCTCAAAGTGGTACAAGTGGTCCAAGTGGTCCAAGTGGTACAAGTCCATCATCTCGTTCAAACACTTTACCTCGTTCAAATACTTCATCTG

**Trento 3** PfMSP1- K1 family partial gene sequence

AGTGGTGCAAGTGCTCAAAGTGGTGCAAGTGCTCAAAGTGGTGCAAGTGCTCAAAGTGGTGCAAGTGCTCAAAGTGGTACAAGTGGTCCAAGTGGTCCAAGTGGTACAAGTCCATCATCTCGTTCAAACACTTTACCTCGTTCAAATACTTCATCTG

**Trento 4** PfMSP1- K1 family partial gene sequence

AGTGGTGCAAGTGCTCAAAGTGGTACAAGTGCTCAAAGTGGTACAAGTGCTCAAAGTGGTACAAGTGGTACAAGTGGTACAAGTGGTCCAAGTGGTACAAGTCCATCATCTCGTTCAAACACTTTACCTCGTTCAAATACTTCATCTG

**Trento 5** PfMSP1- K1 family partial gene sequence

AGTGGTGCAAGTGCTCAAAGTGGTGCAAGTGCTCAAAGTGGTGCAAGTGCTCAAAGTGGTGCAAGTGCTCAAAGTGGTACAAGTGGTCCAAGTAGTCCAAGTGGTACAAGTCCATCATCTCGTTCAAACACTTTACCTAGTTCAAATACTTCATCTGG

**Trento 1** PfMSP1- RO33 family partial gene sequence

GATGCTGTAAGTACTCAAAGTGCTAAAAATCCTCCAGGTGCTACAGTACCTTCAGGTACTGCAAGTACTAAAGGTGCTATAAGATCTCCAGGTGCTGCAAATCC

**Trento 2** PfMSP1- RO33 family partial gene sequence

GATGCTGTAAGTACTCAAAGTGCTAAAAATCCTCCAGGTGCTACAGTACCTTCAGGTACTGCAAGTACTAAAGGTGCTATAAGATCTCCAGGTGCTGCAAATCC

**Trento 3** PfMSP1- RO33 family partial gene sequence

GATGCTGTAAGTACTCAAAGTGCTAAAAATCCTCCAGGTGCTACAGTACCTTCAGGTACTGCAAGTACTAAAGGTGCTATAAGATCTCCAGGTGCTGCAAATCC

**Trento 4** PfMSP1- RO33 family partial gene sequence

GATGCTGTAAGTACTCAAAGTGCTAAAAATCCTCCAGGTGCTACAGTACCTTCAGGTACTGCAAGTACTAAAGGTGCTATAAGATCTCCAGGTGCTGCAAATCC

**Trento 5** PfMSP1- RO33 family partial gene sequence

GATGCTGTAAGTACTCAAAGTGCTAAAAATCCTCCAGGTGCTACAGTACCTTCAGGTACTGCAAGTACTAAAGGTGCTATAAGATCTCCAGGTGCTGCAAATCC

**Trento 1** PfCS partial gene sequence

AGGAAACCAAAACATAAAAAATTAAAGCAACCAGGGGATGGTAATCCTGATCCAAATGCAAACCCAAATGTAGATCCCAATGCCAACCCAAATGTAGATCCAAATGCAAACCCAAATGTAGATCCAAATGCAAACCCAAATGCAAACCCAAATGCAAACCCAAATGCAAACCCAAATGCAAACCCAAATGCAAACCCAAATGCAAACCCAAATGCAAACCCAAATGCAAACCCAAATGCAAACCCAAATGCAAACCCCAATGCAAATCCTAATGCAAACCCAAATGCAAACCCAAATGTAAATCCTAATGCAAATCCAAATGCAAACCCAAATGCAAACCCAAACGCAAACCCCAATGCAAATCCTAATGCAAACCCCAATGCAAATCCTAATGCAAATCCTAATGCAAATCCAAATGCAAATCCAAATGCAAACCCAAATGCAAACCCCAATGCAAATCCTAATGCAAATCCAAATGCAAATCCAAATGCAAACCCAAATGC

**Trento 2** PfCS partial gene sequence

AGGAAACCAAAACATAAAAAATTAAAGCAACCAGGGGATGGTAATCCTGATCCAAATGCAAACCCAAATGTAGATCCCAATGCCAACCCAAATGTAGATCCAAATGCAAACCCAAATGTAGATCCAAATGCAAACCCAAATGCAAACCCAAATGCAAACCCAAATGCAAACCCAAATGCAAACCCAAATGCAAACCCAAATGCAAACCCAAATGCAAACCCAAATGCAAACCCAAATGCAAACCCAAATGCAAACCCCAATGCAAATCCTAATGCAAACCCAAATGCAAACCCAAATGTAAATCCTAATGCAAATCCAAATGCAAACCCAAATGCAAACCCAAACGCAAACCCCAATGCAAATCCTAATGCAAACCCCAATGCAAATCCTAATGCAAATCCTAATGCAAATCCAAATGCAAATCCAAATGCAAACCCAAATGCAAACCCCAATGCAAATCCTAATGCAAATCCAAATGCAAATCCAAATGCAAACCCAAATGC

**Trento 3** PfCS partial gene sequence

AGGAAAGCAGACCATGAGAAATTAAAGCAACCAGGGGATGGTAATCCTGATCCAACAGCGGACCCAAATGTTGATCCCAATGCCAACCCAAATGTAGATCCCAATGCCAACCCAAATGTAGATCCAAATGCAAACCCAAATGCAAATCCAAATGCAAACCCAAATGCAAACCCAAATGCAAACCCAAATGCAAACCCAAATGCAAACCCAAATGCAAACCCAAATGCAAACCTAAATGCAAACCTAAATGCAAACCCCAATGCAAATCCTAATGCAAACCCAAATGCAAACCCAAAGGTAGATCCTAATGCAAACCCAAAGGCAAACCCAAAGGCAAACCCAAGGAGAAACCCCAAAGCAAATCCTAATGCAAACCCCAATGCAAATCCTAATGCAAATCCTAATGGAAATCCAAATGCAAATCCAAATGCAAATCCAAAAGCAAACCCCAATGCAAATCCTAATGCCAATCCAAATGCAAATCCAAATGCAAACCCAAATGC

**Trento 1** PfHRP3 partial gene sequence AGATTATTACACGAAAGTCAAGCACATGCAGGTGATGCCCATCATGCACATCATGTAGCTGATGCTCATCATGCTCACCATGCAGCTAATGCTCACCATGCAGCTAATGCTCACCATGCAGCTAATGCTCATCATGCAGCTAATGCTCATCATGCAGCTAATGCTCATCATGCAGCTAATGCTCACCATGCAGCTAATGCTCACCATGCAGCTAATGCTCACCATGCAGCTAATGCTCATCATGCAGCTAATGCTCACCATGCAGCTAATGCTCACCATGCAGCTAATGCTCATCATGCAGCTAATGCTCACCATGCAGCTAATGCTCACCATGCAGCTAATGCTCACCATGCAGCTAATGCTCACCATGCAGCTGATGCTAATCACGGATTTCATTTTAACCTTCACGATAACAATTCCCATACTTTACATCATGCAAAAGCTAATGCTTGTTTTGATGATTCTCACCATGACGATGCCCACCATGATGGAGCACACCACGACGATGCCCACCATGATGGAGCACACCACGACGATGCCCACCATGATGGAGCACACCACGACGATGCCCACCATGATGGAGCACACCACGATGGAGCACACCATGATGGAGCACACCATAATGCCACTACGCATCACTTACACCCA

**Trento 2** PfHRP3 partial gene sequence

AGATTATTACACGAAAGTCAAGCACATGCAGGTGATGCCCATCATGCACATCATGTAGCTGATGCTCATCATGCTCACCATGCAGCTAATGCTCACCATGCAGCTAATGCTCACCATGCAGCTAATGCTCATCATGCAGCTAATGCTCATCATGCAGCTAATGCTCATCATGCAGCTAATGCTCACCATGCAGCTAATGCTCACCATGCAGCTAATGCTCACCATGCAGCTAATGCTCATCATGCAGCTAATGCTCACCATGCAGCTAATGCTCACCATGCAGCTAATGCTCATCATGCAGCTAATGCTCACCATGCAGCTAATGCTCACCATGCAGCTAATGCTCACCATGCAGCTAATGCTCACCATGCAGCTGATGCTAATCACGGATTTCATTTTAACCTTCACGATAACAATTCCCATACTTTACATCATGCAAAAGCTAATGCTTGTTTTGATGATTCTCACCATGACGATGCCCACCATGATGGAGCACACCACGACGATGCCCACCATGATGGAGCACACCACGACGATGCCCACCATGATGGAGCACACCACGACGATGCCCACCATGATGGAGCACACCACGATGGAGCACACCATGATGGAGCACACCATAATGCCACTACGCATCACTTACACCCA

**Trento 3** PfHRP3 partial gene sequence

AGATTATTACACGAAAGTCAAGCACATGCAGGTGATGCCCATCATGCACATCATGTAGCTGATGCTCATCATGCTCACCATGCAGCTAATGCTCATCATGCAGCTAATGCTCACCATGCAGCTAATGCTCACCATGCAGCTAATGCTCACCATGCAGCTAATGCTCACCATGCAGCTAATGCTCATCATGCAGCTAATGCTCACCATGCAGCTAATGCTCACCATGCAGCTAATGCTCACCATGCAGCTAATGCTCACCATGCAGCTAATGCTCACCATGCAGCTAATGCTCACCATGCAGCTAATGCTCACCATGCAGCTGATGCTAATCACGGATTTCATTTTAACCTTCACGATAACAATTCCCATACTTTACATCATGCAAAAGCTAATGCTTGTTTTGATGATTCTCACCATGACGATGCCCACCATGATGGAGCACACCACGACGATGCCCACCATGATGGTGCACACCACGACGATGCCCACCATGATGGAGCACACCACGATGGAGCACACCATGATGGAGCACACCATGATGGAGCACACCATGATGGAGCACACCATAATGCCACTACGCATCCCTTACACCCA
