# Supplementary material for: Non-imported malaria in Italy: paradigmatic approaches and public health implications following an unusual cluster of cases in 2017
Source: BMC Public Health. 2020 Jun 5;20:857. doi: 10.1186/s12889-020-08748-9 (PMC7275312; doi:10.1186/s12889-020-08748-9)
Supplement: Supplementary file 2 — Additional file 2. Nucleotide sequences from the genetic markers analyzed for the molecular investigations of the putative induced malaria case, Florence 1, and the imported malaria case, Florence 2. [file 12889_2020_8748_MOESM2_ESM.docx]

**Additional file 2.** Nucleotide sequences from the genetic markers analyzed for the molecular investigations of the putative induced malaria case, Florence 1, and the imported malaria case, Florence 2.

**FLORENCE 1** PfMSP1 partial gene sequence

AAATGGTATTAAATGAAGAAGAAATTACTACAAAAGGTGCAAGTGCTCAAAGTGGTGCAAGTGCTCAAAGTGGTGCAAGTGCTCAAAGTGGTGCAAGTGCTCAAAGTGGTGCAAGTGCTCAAAGTGGTGCAAGTGCTCAAAGTGGTGCAAGTGCTCAAAGTGGTGCAAGTGCTCAAAGTGGTACAAGTGGTCCAAGTGGTCCAAGTGGTACAAGTCCATCATCTCGTTCAAACACTTTACCTCGTTCAAATACTTCATCTGGTGCAAGCCCTCCAGCTGATGCAAGCGATTCAGATGCTAAATCTTACGCTGATTTAAAACACAGAGTACGAAATTACTTGTTCACTATTAAAGAACTCAAATATCCCGAACTCTTTGATTTAACCAATCATATGTTAACTTTGTGTGATAATATTCATGGTTTCAAATATTTAATTGATGGATATGAAGAAATTAATGAAT

**FLORENCE 2** PfMSP1 partial gene sequence

AAATGGTATTAAATGAAGAAGAAATTACTACAAAAGGTGCAAGTGCTCAAAGTGGTGCAAGTGCTCAAAGTGGTGCAAGTGCTCAAAGTGGTGCAAGTGCTCAAAGTGGTGCAAGTGCTCAAAGTGGTGCAAGTGCTCAAAGTGGTGCAAGTGCTCAAAGTGGTGCAAGTGCTCAAAGTGGTACAAGTGGTCCAAGTGGTCCAAGTGGTACAAGTCCATCATCTCGTTCAAACACTTTACCTCGTTCAAATACTTCATCTGGTGCAAGCCCTCCAGCTGATGCAAGCGATTCAGATGCTAAATCTTACGCTGATTTAAAACACAGAGTACGAAATTACTTGTTCACTATTAAAGAACTCAAATATCCCGAACTCTTTGATTTAACCAATCATATGTTAACTTTGTGTGATAATATTCATGGTTTCAAATATTTAATTGATGGATATGAAGAAATTAATGAAT

**FLORENCE 1** PfMSP2 partial gene sequence

CAAATGAAGGTTCTAATACTAATAGTGTAGGTGCAAATGCTCCAAATGCTGATACTATTGCTAGTGGAAGTCAAAGGAGTACAAATAGTGCAAGTACTAGTACTACTAATAATGGAGAATCACAAACTACTACTCCTACCGCTGCTGATACCCCTACTGCTACAGAAAGTTCAAGTTCTGGCAATGCACCAAATAAAACAGACGGTAAAGGAGAAGAGAGTAAAAAACAAAA

**FLORENCE 2** PfMSP2 partial gene sequence

CAAATGAAGGTTCTAATACTAATAGTGTAGGTGCAAATGCTCCAAATGCTGATACTATTGCTAGTGGAAGTCAAAGGAGTACAAATAGTGCAAGTACTAGTACTACTAATAATGGAGAATCACAAACTACTACTCCTACCGCTGCTGATACCCCTACTGCTACAGAAAGTTCAAGTTCTGGCAATGCACCAAATAAAACAGACGGTAAAGGAGAAGAGAGTAAAAAACAAAA

**FLORENCE 1** GLURP partial gene sequence

TCAGAACTACATGAAAATGAAGTGGCTCATCCAGAAATTGTTGAAATCGAGGAAGTTATTCCTGAACCAAATCAAAATAACGAATTCCAAGAAATTAATGAAGATGATAAAAGTGCACATATTCAGCATGAAATAGTAGAAGTAGAAGAAATACTTCCAGAAGATGATAAAAATGAAAAAGTTGAACATGAAATAGTAGAAGTTGAAGAAATTCTACCAGAAGATAAAAATGAAAAAGTTCAACATGAAATAGTAGAGGTTGAAGAAATTCTACCAGAAGATAAAAATGAAAAAGTTCAACATGAAATAGTAGAGGTTGAAGAAGTTCTACCAGAAGATGATAAAAATGAAAAAGTTCAACATGATATAGTAGAGGTTGATGAAGTTCTACCAGAAGATGATAAAAATGAAAAAGATCAACATGAAATAGTAGAGGTTGAAGAAATTCTAGCAGAAGATAAAAATGAAAAAGTTCAACATGAAATAGTAGAGGTTGAAGAAATTCTACCAGAAGATGAGGAAAATGAAAAAGTTCAACATGAAATAGTAGAGGTTGAAGAAATTCTACCAGAAGATGATAAAAATGAAAAAGGTCAACATGAAATAGTAGAGGTTGAAGAAATTCTACCAGAAGATAAAAATGAAAAAGTTCAACATGAAATAGTAGAGGTTGAAGAAATTCT

**FLORENCE 2** GLURP partial gene sequence

TCAGAACTACATGAAAATGAAGTGGCTCATCCAGAAATTGTTGAAATCGAGGAAGTTATTCCTGAACCAAATCAAAATAACGAATTCCAAGAAATTAATGAAGATGATAAAAGTGCACATATTCAGCATGAAATAGTAGAAGTAGAAGAAATACTTCCAGAAGATGATAAAAATGAAAAAGTTGAACATGAAATAGTAGAAGTTGAAGAAATTCTACCAGAAGATAAAAATGAAAAAGTTCAACATGAAATAGTAGAGGTTGAAGAAATTCTACCAGAAGATAAAAATGAAAAAGTTCAACATGAAATAGTAGAGGTTGAAGAAGTTCTACCAGAAGATGATAAAAATGAAAAAGTTCAACATGATATAGTAGAGGTTGATGAAGTTCTACCAGAAGATGATAAAAATGAAAAAGATCAACATGAAATAGTAGAGGTTGAAGAAATTCTAGCAGAAGATAAAAATGAAAAAGTTCAACATGAAATAGTAGAGGTTGAAGAAATTCTACCAGAAGATGAGGAAAATGAAAAAGTTCAACATGAAATAGTAGAGGTTGAAGAAATTCTACCAGAAGATGATAAAAATGAAAAAGGTCAACATGAAATAGTAGAGGTTGAAGAAATTCTACCAGAAGATAAAAATGAAAAAGTTCAACATGAAATAGTAGAGGTTGAAGAAATTCT

**FLORENCE 1** PfHRP2 partial gene sequence

ATTTAAATAAGAGATTATTACACGAAACTCAAGCACATGTAGATGATGCCCATCATGCTCATCATGTAGCCGATGCCCATCATGCTCATCATGTAGCCGATGCCCATCATGCTCATCATGTAGCCGATGCCCATCATGCTCATCATGTAGCCGATGCCCATCATGCTCATCATGTAGCCGATGCCCATCATGCTCATCATGCAGCCGATGCCCATCATGCTCATCATGCAGCCGATGCCCATCATGCTCATCATGCAGCTGATGCTCATCATGCTCATCATGCAGCCGATGCCCATCATGCTCATCATGCAGCCGATGCCCATCATGCTCACCATGCAGCTGATGCTCATCACGCTCATCATGCAGCCGATGCCCATCATGCTCATCATGCAGCCTATGCCCATCATGCTCATCATGCATCCGATGCTCATCATGCAGCTGATGCTCACCATGCAGCTCATGCCCATCACGCTCATCATGCAGCTGATGCTCATCATGCAGCCGATGCTCACCATGCAACCGATGCTCATCATGCAGCCGATGCTCACCATGCAGCCGATGCTCACCATGCAGCCGATGCTCACCATGCAACCGATGCTCATCATGCAGCCGATGCTCACCATGCAGCCGATGCTCACCATGCAACCGATGCTCATCACGCTCACCATGCAGCCGATGCTCATCATGCAGCCGCACACCATGCAACTGATGCTCACCATGCAGCCGCACACCATGCAACCGATGCTCACCATGCAGCCGCAC

**FLORENCE 2** PfHRP2 partial gene sequence

ATTTAAATAAGAGATTATTACACGAAACTCAAGCACATGTAGATGATGCCCATCATGCTCATCATGTAGCCGATGCCCATCATGCTCATCATGTAGCCGATGCCCATCATGCTCATCATGTAGCCGATGCCCATCATGCTCATCATGTAGCCGATGCCCATCATGCTCATCATGTAGCCGATGCCCATCATGCTCATCATGCAGCCGATGCCCATCATGCTCATCATGCAGCCGATGCCCATCATGCTCATCATGCAGCTGATGCTCATCATGCTCATCATGCAGCCGATGCCCATCATGCTCATCATGCAGCCGATGCCCATCATGCTCACCATGCAGCTGATGCTCATCACGCTCATCATGCAGCCGATGCCCATCATGCTCATCATGCAGCCTATGCCCATCATGCTCATCATGCATCCGATGCTCATCATGCAGCTGATGCTCACCATGCAGCTCATGCCCATCACGCTCATCATGCAGCTGATGCTCATCATGCAGCCGATGCTCACCATGCAACCGATGCTCATCATGCAGCCGATGCTCACCATGCAGCCGATGCTCACCATGCAGCCGATGCTCACCATGCAACCGATGCTCATCATGCAGCCGATGCTCACCATGCAGCCGATGCTCACCATGCAACCGATGCTCATCACGCTCACCATGCAGCCGATGCTCATCATGCAGCCGCACACCATGCAACTGATGCTCACCATGCAGCCGCACACCATGCAACCGATGCTCACCATGCAGCCGCAC

**FLORENCE 1** PfHRP3 partial gene sequence

TTAATTCAAATAAGAGATTATTACACGAAAGTCAAGCACATGCAGGTGATGCCCATCATGCACATCATGTAGCTGATGCCCATCATGCACATCATGTAGCTGATGCACATCATGCTCACCATGCAGCTAATGCTCACCATGCAGCTAATGCTCACCATGCAGCTAATGCTCACCATGCAGCTAATGCTCATCATGCAGCTAATGCTCACCATGCAGCTAATGCTCATCATGCAGCTAATGCTCACCATGCAGCTAATGCTCACCATGCAGCTAATGCTCACCATGCAGCTAATGCTCACCATGCAGCTAATGCTCACCATGCAGCTAATGCTCACCATGCAGCTAATGCTCACCATGCAGCTGATGCTAATCACGGATTTCATTTTAACCTTCACGATAACAATTCCCATACTTTACATCATGCAAAAGCTAATGCTTGTTTTGATGATTCTCACCATGACGATGCCCACCATGATGGAGCACACCACGACGATGCCCACCATGATGGAGCACACCATGATGGAGCACACCACGACGATGCCCACCATGATGGAGCACACC

**FLORENCE 2** PfHRP3 partial gene sequence

TTAATTCAAATAAGAGATTATTACACGAAAGTCAAGCACATGCAGGTGATGCCCATCATGCACATCATGTAGCTGATGCCCATCATGCACATCATGTAGCTGATGCACATCATGCTCACCATGCAGCTAATGCTCACCATGCAGCTAATGCTCACCATGCAGCTAATGCTCACCATGCAGCTAATGCTCATCATGCAGCTAATGCTCACCATGCAGCTAATGCTCATCATGCAGCTAATGCTCACCATGCAGCTAATGCTCACCATGCAGCTAATGCTCACCATGCAGCTAATGCTCACCATGCAGCTAATGCTCACCATGCAGCTAATGCTCACCATGCAGCTAATGCTCACCATGCAGCTGATGCTAATCACGGATTTCATTTTAACCTTCACGATAACAATTCCCATACTTTACATCATGCAAAAGCTAATGCTTGTTTTGATGATTCTCACCATGACGATGCCCACCATGATGGAGCACACCACGACGATGCCCACCATGATGGAGCACACCATGATGGAGCACACCACGACGATGCCCACCATGATGGAGCACACC
